# Reducing recurrence rates in hiatal hernia repair: Results of a quality improvement study

**DOI:** 10.1007/s11845-024-03743-0

**Published:** 2024-07-18

**Authors:** Laura M. Staunton, Jarlath C. Bolger, Rakesh Ahmed, Waqas T. Butt, John V. Reynolds, Narayanasamy Ravi, Claire L. Donohoe

**Affiliations:** 1 Department of General Surgery, St. James’s Hospital, Dublin, Ireland; 2 Trinity Centre for Health Sciences, St. James’s Hospital and Trinity College, Dublin, Ireland

**Keywords:** Gastropexy, Hiatal Hernia, Paraoesophageal hernia, Recurrence

## Abstract

**Background:**

Patient and procedure factors are considered in the decision-making process for surgical repair of hiatal hernias. Recurrence is multi-factorial and has been shown to be related to size, type, BMI and age.

**Aims:**

This study examined recurrence rates in a single institution, identified areas for improved surgical technique, and re-assessed recurrence following implantation of a quality improvement initiative.

**Methods:**

A retrospective review of patients undergoing hiatal hernia repair surgery between 2018 and 2022 was conducted. Demographics, pre-operative characteristics, intra-operative procedures and recurrence rates were reviewed.

**Results:**

Seventy-five patients from 2018 to 2020 and 34 patients from 2021 to 2022 were identified. The recurrence rate was 21% in 2018–2020, with 14% requiring a revisional procedure. Recurrence and re-operation were subsequently reduced to 6% in 2021 and 2022, which was statistically significant (*p* = 0.043). There was an increase in gastropexy from 21% to 41% following the review (*p* = 0.032), which was mainly reserved for large and giant hernias. Procedural and literature review, alongside gastropexy, can be attributed to recurrence rate reduction.

**Conclusions:**

It is important to educate patients on the likelihood and risk factors of recurrence. A comprehensive review of procedures and a quality improvement program in our facility for hiatal hernia repair is shown to reduce recurrence.

## Introduction

Laparoscopic repair with fundoplication is the approach of choice for treatment of symptomatic hiatal hernias [1]. Hiatal hernias are classified into four types. Type I occurs with herniation through the oesophageal junction. Types II–IV are paraoesphageal (PEH) occurring via the oesophageal hiatus in the diaphragm [1]. In Type II, the gastro-oesophageal junction (GEJ) remains anatomically intact; in Type III, the gastro-oesophageal junction and fundus reside in the hernial sac and in Type IV structures other than the stomach herniate [1, 2]. Historically, repair of PEH hiatal hernias was recommended, in part based on a concern of volvulus and emergency surgery and associated significant mortality [1, 2]. The requirement for emergency surgery during surveillance of PEH has been reported at 1.1% with an associated mortality of 5.4%. This compares to 1.4% mortality for elective surgery [2]. Therefore, in recent decades, hiatal hernia repair surgery is reserved for symptomatic patients [2]. In our facility and as shown in literature, patients present with symptoms of gastroesophageal reflux disease (GORD) or depending on the level of GEJ obstruction: dysphagia, epigastric pain, chest pain, dyspnoea, nausea or vomiting [1, 2]. Assessment of quality of life, alongside investigations, for example, barium swallow, remains important factors in decision making for surgery [2].

This tertiary centre is high-volume for cancer, with up to 100 upper gastrointestinal cancer resections per year, whilst also providing specialist care for benign diseases of the oesophagus [3]. Centralisation of oesophageal and gastric cancer services commenced in England in 2001 to high volume centres [4–6] and in Ireland formally in 2011 [4–6]. In the US, centralisation for PEH repair has occurred in recent years, with the majority of operations performed at high volume centres [6].

Laparoscopic hernia repair has been shown to improve quality of life by relieving gastric and/or pulmonary symptoms [7]. This retrospective review examines the caseload of hiatal hernia repair procedures at a single tertiary centre, reviewing indications for surgery, types of hernias, procedures utilised, urgency of repair, recurrence rates and factors that influence recurrence. Re-herniation can occur with reported recurrence rates in literature varying from 1.2% to 66% [1, 7–9]. Recurrence post hiatal hernia repair can be radiological, endoscopic and/or symptomatic. It has been shown previously that recurrence is associated with larger hernia size at initial presentation, as well as obesity and age [1, 3, 7, 8, 10]. Randomised control trials and SAGES guidelines have suggested gastropexy and mesh as useful techniques to reduce recurrence; however, controversy remains over their use [1, 8, 11, 12]. This study performed a review of recurrence rates over 3 years from 2018 to 2020. Factors that have the potential to cause recurrence were elicited, and areas of improvement were highlighted. A re-review of recurrence rates in 2021 and 2022 was then performed to assess improvement in service.

## Methods

A retrospective, observational study examining indications, procedures, outcomes, and revisions in patients treated with laparoscopic hiatal hernia repair was performed at a single institution over 5 years (Jan 2018–Dec 2022). Ethical approval was obtained (ref: 6675). Data was collated through our online patient records with search for “fundoplasty” and “repair to hernia”. Exclusion criteria included any traumatic diaphragmatic hernias, iatrogenic diaphragmatic hernia’s post-oesphagectomy hernia, fundoplication’s performed at the time of myotomy and any cases involving organ perforation (oesophageal or gastric). Congenital defects and anti-reflux procedures whereby a hiatal hernia was absent were also excluded.

Data has been divided into two groups. The first group of patients underwent hernia repair procedures in 2018–2020 (group 1). Findings from this group were presented in our institution in 2020. Procedural and literature review, a highlight of recurrence rates and introduction of gastropexy were performed. The second group of patients underwent hernia repair procedures in 2021 and 2022 (group 2), following these discussions.

Diagnosis of hiatal hernia were performed via radiological investigations, endoscopy, contrast studies, manometry, pH testing and on occasion at time of repair. Investigations were completed as per patients’ symptoms at presentation and urgency of repair. Acute presentation of hiatus hernia requiring inpatient surgical repair were investigated with CT +—OGD. Elective cases were investigated with OGD + -barium swallow + -manometry + -pH studies. For elective cases, CT was reserved for Type IV hernia for surgical planning. Symptoms include gastro-oesophageal reflux disease (GORD), dysphagia, abdominal pain, cough, vomiting, dyspnoea and chest pain and were associated with size of hiatal hernia. The size of the hernia was denoted by radiology and endoscopy; small (≤ 4cm), moderate (5–7cm), large (8–9cm) and giant (≥ 10cm). Definition of recurrence utilised in this study was a diagnosis via radiological or endoscopic investigations, hiatus hernia greater than 2cm, in symptomatic patients. Recurrence occurred either acutely within the same admission (majority within 1–3 days) or chronically post discharge (majority within 3 years). The outpatient investigations were performed when patients were re-referred, due to symptoms, including GORD, dysphagia and cough.

For continuous variables, the mean and standard deviation were calculated. Percentages were used for nominal. Where the sample size is < 75 (group 1) or < 34 (group 2), this shows that data was not available. STATA software was utilised for statistical analysis. Descriptive analysis was performed using Pearson’s Chi Squared for binary variables and Student’s t-test for continuous. Statistical analysis of recurrence was first performed on group 1 using a logistic regression model. The outcome of recurrence was evaluated against independent exploratory variables including BMI, type, size and age. These variables are associated with recurrence, as previously established in literature. Regression analysis was then utilised to analyse, if there was a statistical difference in recurrence, between groups and if gastropexy made a significant difference to recurrence.

## Results

**Fig. 1:**
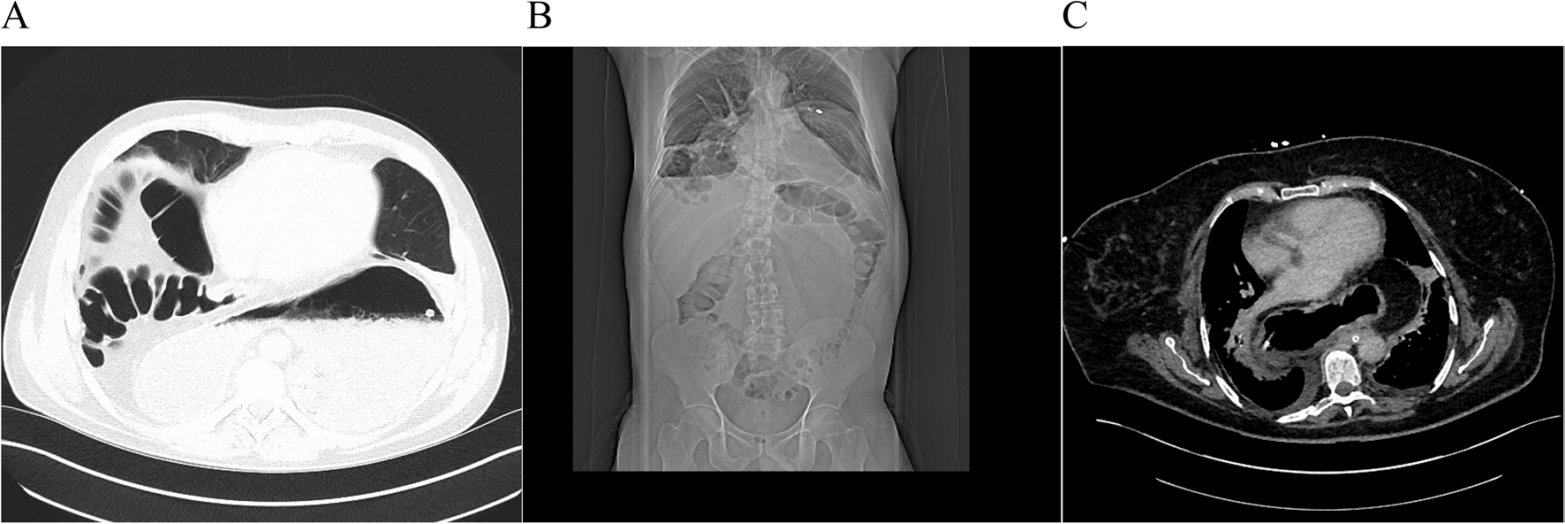
CT images of giant, type IV hernias. The figure shows pre-operative CT images of patients in our review, with type IV giant hiatal hernias of ≥ 10cm in length, with stomach and colon in the thorax

A total of 75 patients between 2018 and 2020 and 35 patients in 2021 and 2022 underwent laparoscopic hiatal hernia repair, with the majority of procedures utilised Nissen fundoplication. Both groups had similar distribution of patient demographics (Table 1). The median deMeester scores were 32.75 and 29.6 in periods 1 and 2, respectively. Of the patients undergoing surgical repair, the majority of patients were in the Type I (32% period 1; 56% in period 2) and Type III (32% in period 1; 33% in period 2). Sixty-three percent of hiatal hernias repaired at our facility were greater than 5cm as measured endoscopically (Table 2). All surgeries were laparoscopic, with two (2.66%) converted to open at time of operation. The majority (88%) of surgeries were performed using a Nissen fundoplication, with partial fundoplication (Toupet) reserved for patients with ineffective motility (Table 3). On review of the two groups, 52% and 44% of surgeries were performed within 1 year of diagnosis, with 39% and 47% within 5 years and 9% and 8% > 6 years (refer to Table 3 for details). Between 2018 and 2020, gastropexy was utilised in 21% of procedures (large/giant hernias), with varying type, number and location of sutures. In 2021 and 2022, gastropexy was utilised in 41% of all cases. It was used in all giant cases, and Type IV repairs and was used in 59% of large hernias in group 2. Mesh was not used in any case. Refer to Fig. 1 for CT images of patient with giant PEH, at our facility.

**Table 1:** Patient demographics

	2018–2020 (Period 1)		2021–2022 (Period 2)		
Characteristic	*N* (%)	Total	*N* (%)	Total	*P* value^1^
No. of patients:	75 (100)		34 (100)		
Sex					
Male	32 (43)	75	16 (47)	34	0.766
Female	44 (58)		18 (53)		
Age (mean ± SD) years	54.2 ± 16.35		55.3 ± 15.5		0.777
BMI (mean ± SD) kg/m^2^	29.4 ± 4.7	55	28.6 ± 3.9	11	0.927
Smoking status					
Current/ex-smoker	26 (38)	68	7(32)	22	0.892
Non-smoker	42 (62)		15 (68)		

^1^Pearson Chi-squared used for binary variables, *t*-test used for continuous variables

**Table 2:** Pre-operative work-up investigations

	2018–2020 (Period 1)		2021–2022 (Period 2)		
Urgency of presentation	*N*^1^ (%)	Total	*N*^1^ (%)	Total	*p* Value^2^
Emergent	11 (15)	75	8 (24)	34	0.347
Urgent	8 (10)		0 (0)		0.066
Elective	56 (75)		26 (76)		0.840
DeMeester score (median)	32.75	33	29.6	12	0.850
Manometry					
Effective motility	25 (60)	43	12 (80)	15	0.129
Ineffective motility	18 (40)		3 (20)		
Indication for surgery					
Paraoesophageal	45 (60)	75	16 (47)	34	0.207
GORD + sliding hiatal hernia	17 (23)		5 (15)		0.007
GORD – hiatal hernia	13 (17)		13 (38)		0.001
Hernia size^3^					
• Giant	• 22 (32)	73	• 3 (9)	34	0.018
• Large	• 10 (14)		• 17 (50)		0.001
• Moderate	• 10 (14)		• 8 (24)		0.184
• Small	• 31 (42)		• 6 (17)		0.011
Hernia type					
• Type I	• 23 (32)	73	• 19 (56)	34	0.012
• Type II	• 18 (25)		• 2 (6)		0.009
• Type III	• 23 (32)		• 11 (33)		0.644
• Type IV	• 9 (12)		• 2 (6)		0.326

^1^Number of patients

^2^Pearson Chi Squared used for binary variables, t-test used for continuous variables

^3^The size of the hernia was denoted by radiology and endoscopy: small (≤ 4cm), moderate (5–7cm), large (8–9cm) and giant (≥ 10cm)

**Table 3:** Surgical details

	2018–2020 (group 1)		2021–2022 (group 2)		
Characteristic	*N*^1^ (%)	Total	*N*^1^ (%)	Total	*P* value
Surgery within 1 year^2^	39 (52)	75	15 (44)	34	0.727
Surgery within 5 years^2^	29 (39)		16 (47)		0.591
Surgery > 6 years^2^	7 (9)		3 (8)		0.932
Nissen Fundoplication	66 (88)		31 (91)		0.865
Partial Fundoplication	9 (12)		3 (19)		0.932
Gastropexy	16 (21)		14 (41)		0.032
• Giant	• 12 (75)	16	• 3 (21)	14	
• Large	• 2 (13)		• 10 (72)		
• Moderate	• 1 (6)		• 1 (7)		
• Small	• 1 (6)		• 0		
Length of stay					
Day case	20 (27)	75	11 (32)	34	0.561
< 5 days	34 (45)		19 (56)		0.160
> 5 days	21 (28)		4 (12)		0.728

^1^Number of patients

^2^Surgery was performed with 1, 5 or > 6 years from diagnosis

Reviewing recurrence for period 1; 8% of all hernia repairs led to an acute recurrence, with the majority (83%) of acute recurrences within 1–3 days of original surgery. Thirteen percent of cases led to chronic re-herniation with the total rate of recurrence of 21% (*n* = 16) (Tables 4 and 5). Eighty-three percent of acute re-herniations were giant at presentation, prior to initial surgery. Sixty-seven percent of acute re-herniation occurred in the over 65s. All acute re-herniations required a revisional procedure. Of the chronic cases that recurred on a repeat presentation (13%), the majority presented with symptoms within 1–3 years (70%) of the original surgery. Investigations with OGD, barium swallow and/or CT were used to detect recurrence of the hiatal hernia. Recurrence symptoms included reflux and dysphagia. Varying types at initial presentation led to recurrence as can be seen in Table 4. Not all chronic re-herniations required a repeat procedure. Reviewing unadjusted and adjusted logistic regression for an association between risk factors of age, BMI, size, type and recurrence, no association can be seen (Tables 5 and 6). Gastropexy was performed in the initial operation in 67% of acute recurrences, all of which were giant hernias.

**Table 4:** Recurrence characteristics

Characteristic	2018–2020 (group 1)		2021–2022 (group 2)	
	*N* (%)	Total	*N* (%)	Total
Re-herniation	16 (21)	75	2 (6)	34
Acute • Presentation (1–3 days) • Presentation (> 1 week)	6 (8)	16	1(3)	2
	• 5 (83)		• 0	
	• 1(17)		• 1	
Chronic • Presentation (1–3 years) • Presentation (4–10 years)	10 (13)	16	1 (3)	2
	• 7 (70)		1 (3)	
	• 3 (30)		0	
Age (> 65)	4 (67)		0	
Age (< 65)	7 (70)		2 (100)	
Re-do procedures	11 (69)	16	2 (100)	2
Gastropexy initial operation (acute)	4 (67)	4	2 (100)	1
• Giant	• 4 (75)		• 0	
• Large	• 0		• 0	
• Moderate	• 0		• 1 (100)	
• Small	• 0		• 0

**Table 5:** Unadjusted logistic regression analysis of recurrence for group 1

Risk factor	*N* (%)	Odds ratio	*p* Value	Lower 95% CL	Upper 95% CL
Type (ref. Type I)					
Type II	6 (38)	1.90	0.381	0.45	7.98
Type III	2 (13)	0.50	0.454	0.08	3.06
Type IV	4 (25)	3.80	0.124	0.69	20.81
Size (ref. small)					
Moderate	3 (19)	1.86	0.453	0.37	9.36
Large	2 (13)	1.08	0.93	0.18	6.46
Giant	5 (31)	1.27	0.722	0.34	4.84
Age	NA	1.00	0.889	0.97	1.04
BMI	NA	1.11	0.176	0.95	1.29

**Table 6:** Adjusted logistic regression analysis of recurrence for group 1

Risk factor	Odds ratio	*p* Value	Lower 95% CL	Upper 95% CL
Type (ref. Type I)				
Type II	1.62	0.586	0.28	9.226
Type III	0.60	0.632	0.075	4.825
Type IV	3.6	0.244	0.42	31.137
Size (ref. small)				
Moderate	4.52	0.147	0.59	34.69
Large	0.67	0.747	0.06	7.83
Giant	1.43	0.69	0.25	8.34
Age	1.01	0.644	0.96	1.06
BMI	1.09	0.255	0.93	1.26

For period 2, the rate of recurrence was 6% (*n* = 2). One patient re-herniated acutely, and one patient recurred within 3 months of initial procedure. Gastropexy and laparoscopic fundoplication was performed initially for both patients. The patient that recurred acutely had a large, Type III hernia prior to initial procedure. The patient that recurred chronically had a moderate, Type I hernia prior to initial procedure. Gastropexy was increased from 21% in period 1 to 41% in period 2 (*p* = 0.032). Regression analysis was performed between recurrence rates of both groups (*p* = 0.043). When gastropexy is included as a predictor variable, recurrence remains statistically significant (*p* = 0.048) Table 7.

**Table 7:** Regression analysis comparing group 1 and group 2

Risk factor	*p* Value	Lower 95% CL	Upper 95% CL
Recurrence	0.043	− 1.515	− 0.24
Recurrence + gastropexy	0.048	− 1.515	− 0.006

## Discussion

In recent decades, hiatal hernia repair surgery is reserved for symptomatic patients, as is the case in our centre [2]. Literature reports indicate up to 66% rate of recurrence post laparoscopic hiatal hernia repair, particularly for PEH [8]. Review of our recurrence rate from 2018 to 2020 revealed that 21% (*n* = 16) of patients experienced symptomatic recurrence, with 14% requiring a re-do procedure. However, group 2, in 2021 and 2022, revealed a significant reduction in recurrence rate (*p*=0.043) and re-do procedures to 6%. Procedural and literature review, in addition to increased gastropexy rates, can be attributed to this.

It has been suggested that recurrences are diagnosed when greater than 2cm and on endoscopic and radiological evaluation [1, 3, 7, 8, 10]. Many hiatal hernia recurrences can be asymptomatic. Braghetto et al. have suggested to consider symptoms, alongside radiological and endoscopic evidence, to diagnose a “true” recurrence [10]. Despite the fact that high recurrence rates exist, hiatal hernia repair procedures have been shown to significantly improve quality of life, even in patients with evidence of recurrence. Reported risk factors requiring a re-do procedure include age (> 65), elevated BMI (> 25), larger size and type (III/IV) of PEH at presentation. Logistic regression analysis of our data from period 1 showed no association between recurrence and BMI, age, type or size at presentation; however, this could reflect our modest sample size. Type IV hernias in this study showed higher odds of recurrence compared to that of other types (OR 3.89, *p*=0.124). As shown in our study and in literature, recurrences are often multi-factorial [1, 3, 7, 8, 10].

Gastropexy as a component of PEH repair, has been reported to reduce recurrence [1, 13–16]. Gastropexy is performed with anterior or posterior sutures and is reported to be most useful for patients, with high risk of recurrence (increased age, elevated BMI, large hernial size) [13–16]. Anterior gastropexy is more commonly utilised [13–17]. Poncet et al. showed, on review of 89 patients, when anterior gastropexy was included during large hiatal hernia repair, without mesh, a significant reduction in recurrence rate, even after 4 years was observed [16]. Yano et al. classifies patients by “anatomy-function-pathology”, showing low recurrence rates when performing mesh repair and gastropexy, on patients deemed at highest recurrence risk, paraoesophageal or mixed hiatal hernias [14]. Pallabazzer et al. have shown during their study, that hiatal hernia repair with complete sac excision, direct closure and use of fundoplication and gastropexy have low recurrence rates (13.1%) and high patient satisfaction (92%) [17]. Gastropexy was increased from group 1 to group 2 in our patient cohort (*p* = 0.032) and led to a significant reduction in recurrence (*p*=0.048). As mentioned previously, patient factors also lead to recurrence, as both recurrences in group 2 included gastropexy, as part of initial procedure.

The use of mesh is controversial. Literature to date supports the potential of mesh to decrease short-medium term recurrence, especially in large and giant repairs [1, 18–21]. However, due to differing hernia shapes and sizes, choice of biological or synthetic mesh and varying surgical techniques, use and benefit of mesh, remains contentious [11, 12, 18–20]. The evidence is better for synthetic mesh; however, complications include erosion, oesophageal stenosis, effusion, pericardial tamponade and chronic inflammation [12]. A 2020 randomised controlled trial by Watson et al. shows few complications with biologic mesh; however, after a 5-year follow-up, mesh repair for large hiatal hernias is not recommended, as reduction in recurrence rates was not found [11]. Other literature supports the use of biological mesh for short-medium term reduction of recurrence rates, especially in larger hernias [1, 19]. Data comparing synthetic to biologic mesh are lacking. Due to the above considerations, mesh was not used at our facility. There are ongoing trials for synthetic and biologic mesh and anterior gastropexy and their usefulness in hiatal hernia repair [20, 21].

Is there a role for benign UGI specialisation in Ireland? In England, reduced mortality rates in high volume cancer specialisation centres in the treatment of benign emergency procedures have been reported [4–6]. Surgeons from high volume oesophageal cancer centres treating emergency PEH and acute re-herniation have extensive experience with complex patients, as well as access to resources. These centres are more likely to treat surgically however not always on first admission [4–6]. Due to an increased risk of mortality of surgery in the acute setting, OGD and nasogastric tube decompression are often initially utilised [5]. When surgical intervention is performed, there is a reduction in postoperative complications (pneumonia and pleural effusions) in high volume centres [5]. Shorter inpatient stays alongside a reduction in postoperative complications were also reported from US high volume hospitals [3]. In our facility, 25% required an urgent or emergent procedure; 72–88% of patients had an inpatient stay of < 5 days. We would contend that benign surgical procedure like hiatal hernia repair, should be performed in high volume centres, with continuous audit and targeted quality improvement [4–6].

In conclusion, recurrence post hiatal hernia repair is multi-factorial. Procedural and literature review, in addition to a standardised gastropexy procedure, has been shown to reduce recurrence rates in our facility (*p*=0.043). During education and consent of hiatal hernia repair with patients, including discussion around recurrence rates, it is worth considering that size at presentation, prompt surgery scheduling and repair improve symptoms regardless of recurrence.

## Data Availability

Data supporting the findings of this study are available from the corresponding author, upon reasonable request.
